# The use of dexamethasone in the treatment of COVID-19

**DOI:** 10.1016/j.amsu.2020.07.004

**Published:** 2020-07-09

**Authors:** Mohammed Lester, Ali Sahin, Ali Pasyar

**Affiliations:** University College London Medical School, 74 Huntley Street, Bloomsbury, London, WC1E 6DE, UK

Dear Editor,

After reading around regarding the state of knowledge surrounding the COVID-19 outbreak, we were intrigued by the lack of a general consensus on what constituted effective anti-viral therapy to treat an infection of COVID-19. In this letter, we plan to highlight the recent recommendation of Dexamethasone as a treatment for COVID-19 patients undergoing respiratory support, the pharmacological basis for the treatment and its potential usage in the healthcare system.

Although recently there is no current treatment for COVID-19, there have been clinical trials underway to determine potential treatments using existing drugs. The Randomised Evaluation of COVid-19 thERapY (RECOVERY) Trial currently underway in the UK, announced on the 16th June 2020 that dexamethasone had been shown to cause significant improvement in the outcomes of COVID-19 patients undergoing respiratory support [1].

COVID-19 in some patients can lead to the development of pneumonia [2]. The fluid accumulation in COVID-19 associated pneumonia is a result of inflammation brought about by the secretion of inflammatory chemokines, such as TNF-α, released by immune cells, such as neutrophils [2]. This can eventually lead to Acute respiratory distress syndrome (ARDS) which is the primary contributor to mortality in COVID-19 positive patients [2]. Dexamethasone is glucocorticoid that acts as a synthetic version of the naturally occurring hormone cortisol. It has the same anti-inflammatory effects of cortisol, namely the inhibition of the release of inflammatory chemokines by immune cells [3]. This has the potential to reduce the inflammation in the lungs thus improving patient prognosis by decreasing the severity of ARDS.

The use of dexamethasone was recommended by the four chief medical officers of the UK on the 16^th^ June 2020 for COVID-19 positive patients receiving respiratory support, based on the interim data published by the RECOVERY trial [4]. The trial has shown that for ventilated patients dexamethasone treatment is able to reduce deaths by 35% (rate ratio 0.65 [95% confidence interval 0.48 to 0.88]; p = 0.0003) and in patients receiving oxygen therapy, deaths are reduced by 20% (0.80 [0.67 to 0.96]; p = 0.0021) [1]. Finally, it was also found that there was no improvement in patients who did not require respiratory support 1.22 [0.86 to 1.75; p = 0.14); thus supporting the recommendations of the chief medical officer for dexamethasone use only in patients undergoing respiratory support [1].

The RECOVERY trial is a well-designed study with over 6000 patients recruited across 170 NHS trusts for the dexamethasone arm of the trial [1]. The study was conducted in accordance with the principles of International Conference on Harmonisation Guidelines for Good Clinical Research Practice (ICH-GCP) [5]. In this regard, a number of steps were taken to ensure observer bias was limited. Despite being an open label study, access to the outcomes of the investigation were not made available to any members of the research teams, Steering Committee or patients during its progression. Additionally, while having the interim trial results monitored by an independent data monitoring committee, it was also ensured that the funding received for the study did not involve any entities linked to the pharmaceutical industry, which could pose a potential source of bias [5].

The primary outcome being measured in this study is mortality at 28 days after first randomisation [5]. Although the study protocol fails to account for all-cause mortality over COVID-specific mortality, it should be noted that all-cause mortality is more useful as specific COVID-19 mortality may be more subjective. In addition to this, only serious adverse events which were deemed to be related to the intervention were noted [5]. The reporting of only serious adverse events means that there is no accurate data on the burden of intervention. Some patients could have experienced minor intervention related adverse events over prolonged periods of time which would not be recorded due to the study's design.

To summarise, RECOVERY trial is a multi-arm trial looking at various interventions for COVID-19. The dexamethasone arm of the trial shows concrete evidence for the use of the drug in patients receiving respiratory support. So far, the, RECOVERY trial is the strongest available evidence for the use of glucocorticoids in the treatment of COVID-19 and has resulted in dexamethasone being licenced by NHS England for use in patients with COVID-19 who are undergoing respiratory support [4]. We look forward to a more comprehensive picture emerging of the treatments for COVID-19 as the RECOVERY trial progresses over the coming months.

## Ethical approval

Ethical approval was not required for this letter. All data used is publicly accessible.

## Funding

There were no external sources of funding for this research.

## Author contribution

Mr Mohammed Lester was the lead author of this letter.

Mr Ali Sahin and Mr Ali Pasyar were also authors on this article and helped with data analysis and rewriting parts of the article.

## Registration of research studies

1. Name of the registry: N/A.

2. Unique Identifying number or registration ID: N/A.

3. Hyperlink to your specific registration (must be publicly accessible and will be checked): N/A.

## Guarantor

We, Mr Mohammed Lester, Mr Ali Sahin and Mr Ali Pasyar accept full responsibility for this commentary article.

## Consent

Consent was not required for this letter. All data used is publicly accessible.

## Declaration of competing interest

We, Mr Mohammed Lester, Mr Ali Sahin and Mr Ali Pasyar, do not report any conflicts of interest in the writing of this letter.
